# Observation of Nociceptive Processing: Effect of Intra-Epidermal Electric Stimulus Properties on Detection Probability and Evoked Potentials

**DOI:** 10.1007/s10548-020-00816-y

**Published:** 2021-01-18

**Authors:** Boudewijn van den Berg, Jan R. Buitenweg

**Affiliations:** Biomedical Signals and Systems, Technical Medical Centre, University of Twente, PO Box 217, 7500 AE Enschede, The Netherlands

**Keywords:** Electroencephalography, Psychophysics, Evoked potential, Detection threshold, Detection probability, Intra-epidermal electric stimulation, Nociception, Nociceptive Processing, Peripheral nerve fiber recruitment, Central synaptic summation, Habituation

## Abstract

Monitoring nociceptive processing is a current challenge due to a lack of objective measures. Recently, we developed a method for simultaneous tracking of psychophysical detection probability and brain evoked potentials in response to intra-epidermal stimulation. An exploratory investigation showed that we could quantify nociceptive system behavior by estimating the effect of stimulus properties on the evoked potential (EP). The goal in this work was to accurately measure nociceptive system behavior using this method in a large group of healthy subjects to identify the locations and latencies of EP components and the effect of single- and double-pulse stimuli with an inter-pulse interval of 10 or 40 ms on these EP components and detection probability. First, we observed the effect of filter settings and channel selection on the EP. Subsequently, we compared statistical models to assess correlation of EP and detection probability with stimulus properties, and quantified the effect of stimulus properties on both outcome measures through linear mixed regression. We observed lateral and central EP components in response to intra-epidermal stimulation. Detection probability and central EP components were positively correlated to the amplitude of each pulse, regardless of the inter-pulse interval, and negatively correlated to the trial number. Both central and lateral EP components also showed strong correlation with detection. These results show that both the observed EP and the detection probability reflect the various steps of processing of a nociceptive stimulus, including peripheral nerve fiber recruitment, central synaptic summation, and habituation to a repeated stimulus.

## Introduction

A major challenge in the development of pain biomarkers is the complex nature of the pain experience, as it is determined by a significant amount of supra-spinal processing of the initial sensory input (Apkarian et al. 2005). Developing methods to accurately measure the relation between a well-defined sensory input, brain activation, and pain perception might 1 day lead to more objective mechanism-based pain biomarkers. A well-defined nociceptive sensory input can be generated by preferential stimulation of nociceptive Aδ afferents in the skin using intra-epidermal electric stimulation (Inui et al. 2002). This technique has been shown to preferentially activate nociceptive afferents when applied at less than two times the detection threshold (Mouraux et al. 2010; Poulsen et al. 2020). Recently, we developed a method to concentrate stimulation around this (drifting) detection threshold, and measure stimulus–response pairs and evoked potentials in response to these nociceptive stimuli (van den Berg et al. 2020).

Based on acquired stimulus–response pairs, a psychometric function for the detection probability can be determined which is characterized by a detection threshold and a slope. Recent research has demonstrated that the detection threshold can be used to observe both short-term and long-term effects of experimental pain conditioning. Conditioned pain modulation by immersion of one foot in ice water resulted in a direct increase of the detection threshold of single-pulse intra-epidermal stimuli (Doll et al. 2014). On the other hand, 1-h application of an 8% capsaicin patch resulted in a long-term increase of the detection threshold to intra-epidermal stimuli (Doll et al. 2016a, b). More specifically, detection thresholds to single-pulse intra-epidermal stimuli were significantly increased on days 2 to 7 following capsaicin application, while detection thresholds to double-pulse intra-epidermal stimuli were significantly increased on days 7 to 28 after capsaicin application. The difference between both stimulus types was that by using two or more pulses, we also observed the effect of temporal summation on nociceptive processing, which lead to a significant decrease of the detection threshold and increase of the slope in the case of double-pulse intra-epidermal stimuli (Doll et al. 2016a, b).

Centering stimulus amplitudes around the detection threshold allows for the measurement of evoked potentials in response to nociceptive intra-epidermal stimulation. Earlier studies showed that intra-epidermal stimulation at twice the detection threshold results in an evoked potential waveform that is sensitive to experimental pain conditioning such as the intra-epidermal injection of capsaicin (Liang et al. 2016) and high-frequency stimulation (Manresa et al. 2018). Typically this evoked potential included an early contralateral negative peak around 150 ms referred to as the N1 (Mouraux 2014), a central negative peak observed between 130 and 150 ms (Liang et al. 2016) or 220–230 ms (Mouraux et al. 2014), and a central positive peak between 290 and 330 ms (Liang et al. 2016) or 360–370 ms (Mouraux et al. 2014). This difference in latencies might be partly explained by the difference in filter settings used in both studies. Nevertheless, a systematic evaluation of the influence of filter settings on intra-epidermal evoked potential waveforms and topographies has not been done so far. Intra-epidermal evoked potential waveforms were also shown to be affected by the number of pulses, as the N1, N2 and P2 were shown to be larger and more reliable when multiple electric current pulses were applied, while the evoked potential latencies and response times remained the same (Mouraux et al. 2014). However, an evaluation of the influence of the interval between those pulses on the resulting evoked potential was not done yet.

After a technical pilot study (van den Berg et al. 2020), we designed a new study with the combined measurement of detection thresholds and evoked potentials to accurately quantify the effect of intra-epidermal stimulus properties, i.e. number of pulses and inter-pulse interval, on both outcome measures in healthy individuals. More specifically, we wanted to (1) confirm the presence of previously observed EP components in response to intra-epidermal electric stimulation, (2) determine at which scalp locations and which latencies the observed EP components are maximal and (3) analyze in detail how these components and detection thresholds are influenced by intra-epidermal stimulus properties in healthy subjects, using (generalized) linear mixed regression models. After exploring the effect of various filter settings on the EP waveform, we investigated which model most effectively captures the data and studied the effect of detection, pulse amplitudes, trial number and the interaction between detection and trial number. In this way, we aimed to obtain new insights and directions for the design of future studies employing this method to study alterations in nociceptive function.

## Methods

The experiments presented in this work include measurements of the detection threshold and the EEG with respect to intra-epidermal stimuli on a single occasion at the University of Twente, the Netherlands. All experiments were approved by the local Medical Review and Ethics Committee and in accordance with the declaration of Helsinki.

### Participants

A total of 30 healthy participants (20 males and 10 females, age 23.0 ± 3.4, 4 left-handed) were included in this study. To be included, participants had to have an age between 18 and 40 years old. Exclusion criteria were skin abnormalities at the site of stimulation, diabetes, implanted stimulation devices, pregnancy, usage of analgesics within 24 h before the experiment, the consumption of alcohol or drugs within 24 h before the experiments, pain complaints at the time of the experiment, a medical history of chronic pain or any language problems that would impede communication with the participant. All participants provided written informed consent and received a monetary compensation of €20 for participation in the experiment.

### Stimuli

**Fig. 1:**
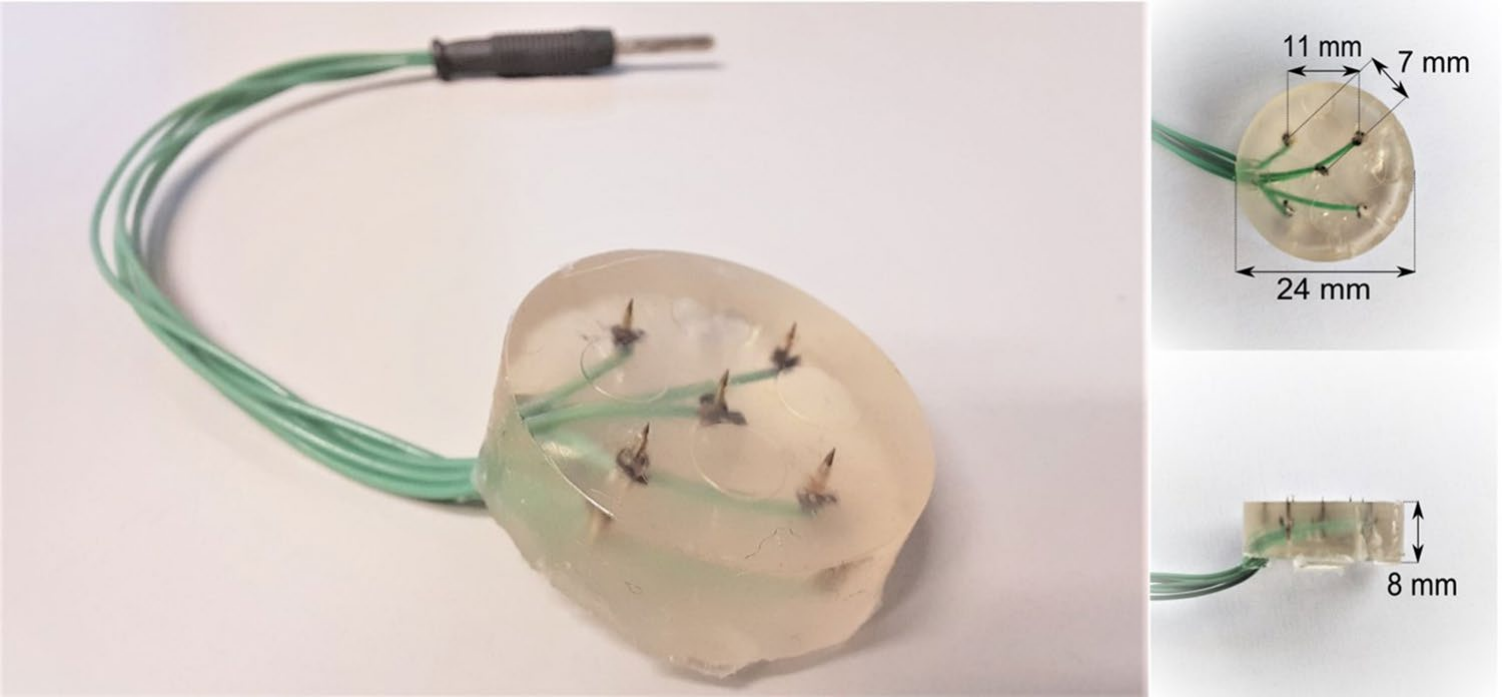
Electrode for intra-epidermal stimulation, consisting of an array of 5 inter-connected microneedles embedded in a flexible layer of silicone

Stimuli consisted of square-wave electrical current pulses generated by a constant current stimulator (NociTRACK AmbuStim, University of Twente, Enschede, The Netherlands) and were applied intra-epidermal to achieve preferential activation of Aδ-fibers (Inui et al. 2002; Mouraux et al. 2010). Stimulation was applied using a custom made electrode consisting of 5 inter-connected microneedles (Fig. 1). A previous validation study of this electrode showed that stimulation resulted in a sharp pricking sensation (Steenbergen 2012).

A previous study measuring the detection probability and detection thresholds in response to intra-epidermal stimulation observed a larger detection probability than would be expected based on probability summation for double-pulse stimuli with inter-pulse intervals on a range of 10 to 100 ms, which suggests that such stimuli are amplified by a facilitatory mechanism (Doll et al. 2016a, b). In this study, we aimed to reproduce this increased detection probability of double-pulse stimuli, and to observe if this is related to an increase of evoked potential. As we wanted to remain well below the inter-pulse interval at which each pulse is observed individually of 200 ms (Lee et al. 2009) and well above the time required for nerve repolarization we used inter-pulse intervals of 10 and 40 ms. As such, a total of three different settings was used:

- A single 210 µs pulse.
- A double 210 µs pulse with an inter-pulse interval of 10 ms.
- A double 210 µs pulse with an inter-pulse interval of 40 ms.

### Procedure

**Fig. 2:**
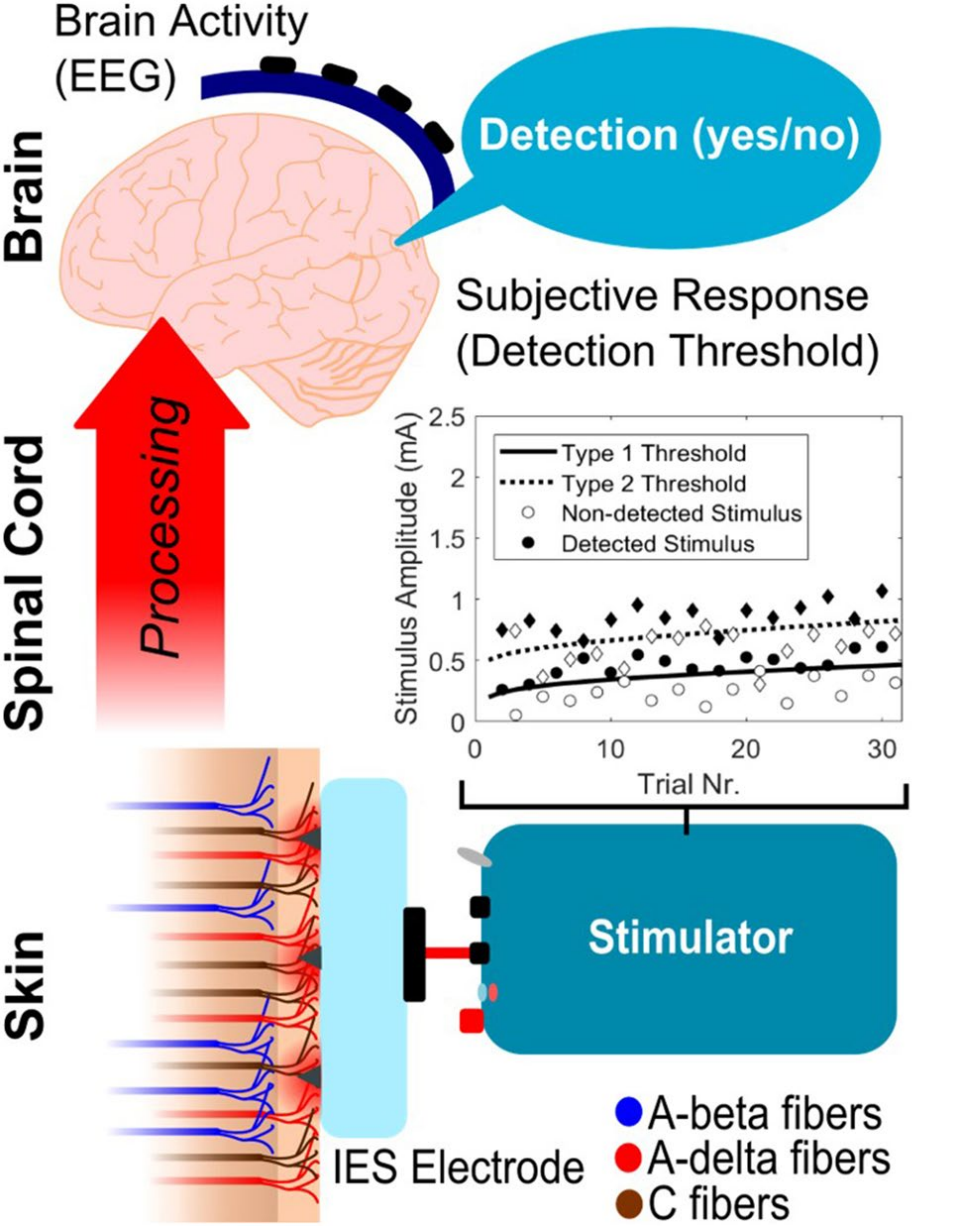
We attempt to characterize nociceptive processing by simultaneously measuring the effect of intra-epidermal stimulus properties on psychophysical detection probability and brain evoked potentials. Multiple stimulus types (i.e. with a different number of pulses or inter-pulse interval) are generated with a varying amplitude close to the detection threshold. Stimuli preferentially activate nociceptive nerve fibers by using a specially designed needle electrode which protrudes into the epidermis of the skin using microneedles. Subject responses (detected or non-detected) and EEG corresponding to the applied stimuli are acquired and analyzed using (generalized) linear mixed regression to quantify the effect of stimulus properties on detection probability and evoked potentials

Participants were seated in a comfortable chair and instructed to focus on the electrode. First, their initial detection threshold was approximated by a standard staircase procedure without inversion with a step size of 0.025 mA. Subsequently, nociceptive detection thresholds were tracked simultaneously for the 3 stimulus types on 30 participants, with a total of 150 stimuli per stimulus type per participant. Participants were instructed to press and hold a button, and shortly release the button as soon as they felt a sensation that they ascribe to the application of a stimulus. While the button was pressed, the stimulator applied stimuli to the participant. Stimulus amplitudes were chosen according to an adaptive staircase procedure designed to converge towards and track a time-dependent psychophysical threshold (Doll et al. 2015). A set of five equidistant amplitudes with a step size of 0.025 mA was defined around the initial detection threshold, from which the next stimulus was randomly selected. A stimulus was identified as detected if the participant released the button within one second after the stimulus, and otherwise considered non-detected. This reaction time was measured internally by the stimulator as the time between stimulus onset and button release, with a resolution 35 microseconds. The set of equidistant amplitudes was decreased by 0.025 mA if a stimulus was detected and increased by 0.025 mA if a stimulus was non-detected. Subsequently, the next stimulus was selected from the updated set of equidistant amplitudes and applied after a uniformly randomized interval of 4.3 to 5.3 s. This procedure was repeated until the end of the experiment (Fig. 2).

### Electroencephalography

The scalp EEG was continuously recorded with a sampling rate of 1024 Hz using a REFA amplifier (TMSi B.V., Oldenzaal, the Netherlands) at 128 Ag/AgCl electrodes with a common average reference. Electrodes were placed on the scalp according to the international 10/5 system (Oostenveld and Praamstra 2001) and additional leads were placed on the earlobes. The participants were asked fix their gaze at a spot on the wall and blink as few times as possible while they pressed the response button and hence received stimuli. Participants with excessive electrode impedance (> 5 channels with > 20 kΩ) were excluded. Therefore, a total of 25 participants (16 males and 9 females, age 23 ± 3.6, 1 left-handed) were used for analysis.

EEG data was pre-processed using FieldTrip (Oostenveld et al. 2011), a Matlab toolbox for EEG and MEG signal processing. Contamination of the EEG by eye-blinks was corrected using an independent component analysis algorithm (Delorme et al. 2007). Subsequently, epochs with excessive EMG activity or movement artefacts were removed by visual inspection. The first 15 epochs were removed as no reliable estimate of the detection threshold was available for those trials. Furthermore, epochs in which the stimulus amplitude exceeded two times the tracked individual detection threshold were excluded from analysis. Based on earlier observations that opercular sources are first activated contralateral with respect to stimulation and lateralized afterwards (Garcia-Larrea et al. 2003), the channel topography for left-handed subjects was inverted such that uneven numbers correspond to the contralateral side and even numbers to the ipsilateral side with respect to stimulation.

Epochs for EP analysis were extracted from the EEG using a window ranging from 0.5 s before until 1.0 s after the stimulus. To compare EP waveforms in the current study, with those previously observed in literature in response to intra-epidermal stimulation, grand average waveforms and topographies at Cz (average reference) and T7-Fz, bandpass filtered at 0.1 to 40 Hz, 0.5 to 45 Hz and 0.1 to 30 Hz and baseline corrected, were set side by side. Further analyses were performed using the waveforms bandpass filtered at 0.1 to 40 Hz to minimize signal loss and the potential bias induced by the high-pass filter (Acunzo et al. 2012).

Average latencies of three peaks in the EP were defined as follows. A first negative peak (N1), was defined as the most negative peak at T7-Fz between 130 and 170 ms after stimulus onset. A second negative peak (N2) was defined as the most negative peak at Cz between 170 and 300 ms after stimulus onset. Lastly, a positive peak (P2) was defined as the most positive peak at Cz between 300 and 500 ms after stimulus onset. To check if each of those peaks coincide with global peaks of EEG activity, peak latencies were compared with the butterfly plot and the global field power (Lehmann and Skrandies 1980).

To systematically study on which locations the N1, N2 and P2 are best observed, the SNR was computed for each channel, where SNR was defined as in (1), where $S(t)$ denotes the grand average potential at latency $t$ and ${\sigma }_{baseline}$ denotes the standard deviation of the grand average from − 0.5 to − 0.3 s with respect to stimulus onset.

1 $$SNR= \frac{|S\left(t\right)|}{{\sigma }_{baseline}}$$

Subsequently, grand average EP waveforms were computed at derivations with a maximum SNR: T7-F4 at N1 and N2, and CPz-A1A2 at P2. The effect of intra-epidermal stimulus properties on these EP waveforms was studied using linear mixed regression, which is further outlined in “Effect of Intra-Epidermal Stimulus Properties on Evoked Potential” section.

### Effect of Intra-Epidermal Stimulus Properties on Detection Probability

Statistical analysis of stimulus–response pairs was performed in R with the lme4 toolbox (Bates et al. 2015). The effect of stimulus properties on the detection probability was estimated using logistic generalized linear mixed regression using a statistical model selected using the procedure outlined in “Model Selection” section. The track of individual and group level thresholds was estimated by performing generalized mixed regression over a moving window of 30 stimulus–response pairs. Subsequently, generalized mixed regression was performed over the entire dataset to establish accurate estimates of effect size and significance. The trial number was centered and scaled to speed up the model estimation process. Estimates of the threshold and slope were obtained using the estimated effect sizes, and corresponding standard errors were approximated using the Delta procedure (Faraggi et al. 2003; Moscatelli et al. 2012). Effect significance was assessed using type III Wald Chi-square statistics with a two-tailed test. As 26 tests are performed in this article, the significance level was set to $0.025/26\approx 0.001$ after Bonferroni correction.

### Effect of Intra-Epidermal Stimulus Properties on Evoked Potential

Statistical analysis of EEG data was performed in MATLAB 2017b (MathWorks, Inc.). The effect size of stimulus properties was evaluated using linear mixed regression (Van den Berg and Buitenweg 2018). Regression parameters were computed for every point in time at CPz-A1A2 and T7-F4 using a statistical model selected using the procedure oultined in “Model Selection” section. Model variables were centered and scaled to speed up the estimation progress. Subsequently, effect sizes and their corresponding *t*-values were estimated for every point in time by optimization of the restricted maximum likelihood. At component latencies (153, 213 and 418 ms) significance of the effect sizes was assessed using the *t-*statistic with a two-tailed test using Satterthwaite’s method for estimation of the degrees of freedom. Similar to the previous section, the significance level was set to 0.001 after Bonferroni correction.

### Model Selection

We compared several models for statistical analysis of the detection probability and EEG, based on the Akaike information criterion (AIC) (Akaike 1974) and the Bayesian information criterion (BIC) (Schwarz 1978). For both criteria a lower value indicates a better model fit, while accounting for overfitting. In the case that the AIC and BIC values did not agree on the same model, the value of the BIC was used to select a statistical model for further analyses.

The first model assumed that neurophysiological activity of both pulses was integrated by temporal summation, where the neurophysiological activity generated as a result of the first pulse amplitude (in mA, denoted by PU1) is summed with activity generated as a result of the second pulse amplitude with either 10 ms IPI (in mA, denoted by PU2_10_) or 40 ms IPI (in mA, denoted by PU2_40_). Furthermore, this signal could decrease with respect to the trial number (denoted by TRL) due to habituation effects.

The resulting generalized linear mixed regression model for computing detection probability in A was compared to models using a direct combination of the experimental parameters in B, C and D, where the response was modeled based on the stimulus amplitude (in mA, denoted by AMP), stimulus type (denoted by TYP) and the trial number. The random effect structure was grouped by subject (denoted by S) and included all model terms. An unstructured covariance structure was used to model the random effects. Models are written in Wilkinson notation (Wilkinson and Rogers 1973), where random effects are written in between brackets and ‘| S’ denotes that the random effects are grouped by subject.

A $$ln\left(\frac{{P}_{d}}{1-{P}_{d}}\right)\sim 1+PU1+PU{2}_{10}+PU{2}_{40}+TRL+\left(1+PU1+PU{2}_{10}+PU{2}_{40}+TRL \right| S)$$

B $$ln\left(\frac{{P}_{d}}{1-{P}_{d}}\right)\sim 1+AMP*TYP*TRL+\left(1+AMP*TYP*TRL \right| S)$$

C $$ln\left(\frac{{P}_{d}}{1-{P}_{d}}\right)\sim 1+AMP*TYP+TRL+\left(1+AMP*TYP+TRL \right| S)$$

D $$ln\left(\frac{{P}_{d}}{1-{P}_{d}}\right)\sim 1+AMP+TYP+TRL+\left(1+AMP+TYP+TRL \right| S)$$

The first linear mixed regression model for analyzing EEG activity was based on the temporal summation model in A, but included a term for additional brain activity evoked by stimulus detection which could increase or decrease with respect to the trial number (denoted by the interaction TRL*D). The resulting model in E was compared to the models based on combinations of experimental parameters in F, G, H and I at the P2 latency (414 ms). The random effect structure was grouped by subject and included all model terms. A diagonal covariance structure was used to model the random effects.

E $${U}_{EEG}\sim 1+PU1+PU{2}_{10}+PU{2}_{40}+TRL*D+\left(1+PU1+PU{2}_{10}+PU{2}_{40}+TRL*D \right| S)$$

F $${U}_{EEG}\sim 1+AMP*TYP*TRL*D+\left(1+AMP*TYP*TRL*D \right| S)$$

G $${U}_{EEG}\sim 1+AMP*TYP*D+TRL*D+\left(1+AMP*TYP*D+TRL*D \right| S)$$

H $${U}_{EEG}\sim 1+AMP*TYP+TRL*D+\left(1+AMP*TYP+TRL*D \right| S)$$

I $${U}_{EEG}\sim 1+AMP+TYP+TRL+D+\left(1+AMP+TYP+TRL+D \right| S)$$

## Results

### Model Selection

A functional model was compared to models directly based on the experimental parameters for statistical analysis of the detection probability and the EEG. Model AIC and BIC values are shown in Table 1. For the detection probability, the experiment-based model B including all effects resulted in the lowest AIC. For EEG data, the functional model E resulted in the lowest AIC. For both the detection probability and the EEG data, the functional models A and E resulted in the lowest BIC.

**Table 1:** AIC and BIC values for comparison of the functional models (A and E) with various experiment-based models

Detection probability			EEG		
Model	AIC	BIC	Model	AIC	BIC
A	13,444	**13,591**	E	**25,069**	**25,177**
B	**13,393**	14,053	F	25,389	25,575
C	13,413	13,670	G	25,107	25,314
D	14,015	14,161	H	25,105	25,241
			I	25,122	25,215

A lower AIC or BIC value indicates a better model fit while accounting for the number of parameters in the model. Values in bold indicate the model with the lowest AIC or BIC

### Effect of Intra-Epidermal Stimulus Properties on Detection Probability

**Fig. 3:**
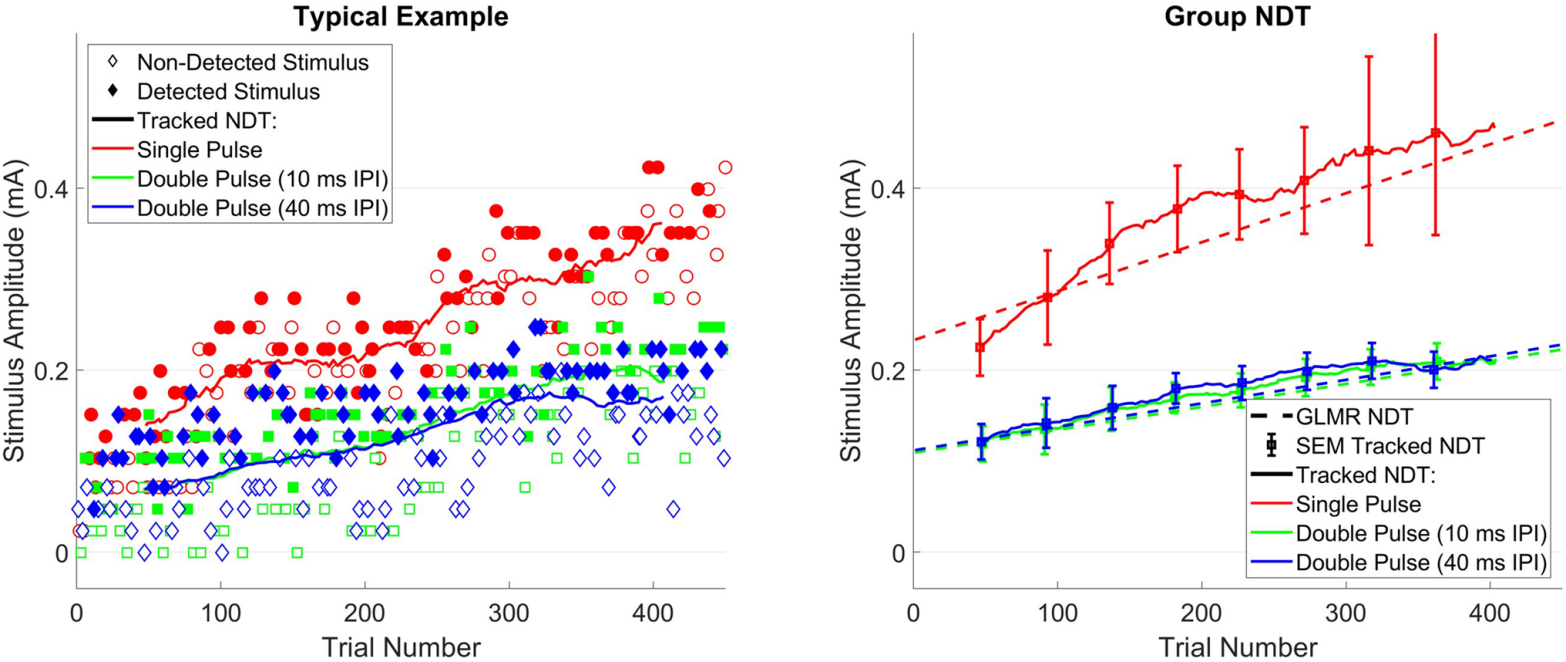
A typical example of tracked nociceptive detection thresholds (NDTs) (left) and group level NDTs (right). Both group level detection thresholds computed over a time window of 30 stimuli (tracked NDT) and computed over the entire experiment (GLMR NDT) are shown. The standard error of the mean (SEM) of tracked thresholds is indicated by bars. For each NDT, it can be observed that the threshold increased over time and that the threshold for double pulse stimuli was much lower. However, there was no difference in detection threshold with respect to inter-pulse interval (IPI)

Subjects detected on average 46.7% of all applied stimuli with a reaction time of 546 ± 161 ms. A typical example of a resulting tracked NDT in a single subject is shown on the left side of Fig. 3. For each stimulus type, the NDT increased over time. Both thresholds for double pulse stimuli were almost equal, i.e. there was no difference of NDT with respect to the inter-pulse interval in this subject. Similar results are shown in the group level thresholds on the right in Fig. 3, computed using the GLMR model over a 30 trial moving window (continuous lines) and over the entire dataset (dotted lines). Group level NDTs computed over the entire dataset increased over the trials and remained within the standard error of the mean (SEM) of NDTs computed over a 30 trial moving window. Once again, no difference was seen between NDTs of double pulse stimuli with 10 ms inter-pulse interval and those with 40 ms inter-pulse interval.

Observations in Fig. 3 are supported by effect sizes and significances in Table 2. There was a significant positive effect size for each of the pulse amplitudes, indicating an increase in detection probability with respect to the pulse amplitudes. There was a significant negative effect size of trial number, indicating a decrease of detection probability with respect to the trial number. In Table 3, it is shown that there was indeed a significantly lower detection threshold (p < 0.001) and steeper slope (p < 0.001) for both types of double-pulse stimuli in comparison with a single-pulse stimulus. However, there was no significant difference in detection threshold or slope between double pulse stimuli with 10 or 40 ms inter-pulse interval.

**Table 2:** Effect of stimulus properties on the detection probability, computed using GLMR

Stimulus property	Effect size	95% Confidence interval	Effect χ^2^	Effect p
(Intercept)	− 3.70	[− 4.32, − 3.09]	139.61	** < .001**
Pulse 1 (PU1)	10.43	[7.14, 13.72]	38.67	** < .001**
Pulse 2, 10 ms IPI (PU2_10_)	11.83	[9.03, 14.63]	68.38	** < .001**
Pulse 2, 40 ms IPI (PU2_40_)	11.31	[8.61, 14.01]	67.40	** < .001**
Trial number (TRL)	− 0.0056	[− 0.0074, − 0.0038]	37.69	** < .001**

All effect sizes and confidence intervals were rescaled to physical units (Int.: -, PU1: mA^−1^, PU2_10_: mA^−1^, PU2_40_: mA^−1^, TRL: trial^−1^). Significance was assessed using type-III Wald Chi-square statistics. All tested stimulus properties have a significant effect on the detection probability. The detection probability decreases with respect to trial number and increases with respect to the amplitude of the first pulse and of the second pulse with either 10 ms or 40 ms IPI

**Table 3:** Detection thresholds (in mA) and slopes (in mA^−1^) per stimulus type

Stimulus Type	Threshold	95% Confidence Interval	Slope	95% Confidence Interval
Single-pulse	0.35	[0.33, 0.52]	10.43	[7.20, 13.66]
Double-pulse, 10 ms IPI	0.17***	[0.14, 0.20]	22.26***	[17.15, 27.37]
Double-pulse, 40 ms IPI	0.17***	[0.14, 0.20]	21.74***	[16.42, 27.06]

There is a significantly (p < 0.001, indicated by ***) lower detection threshold and steeper slope for each type of double-pulse stimulus in comparison to the detection threshold of single-pulse stimuli. There was no significant difference in detection threshold or slope between both types of double-pulse stimuli

### Evoked Potential in Response to Intra-Epidermal Stimuli

**Fig. 4:**
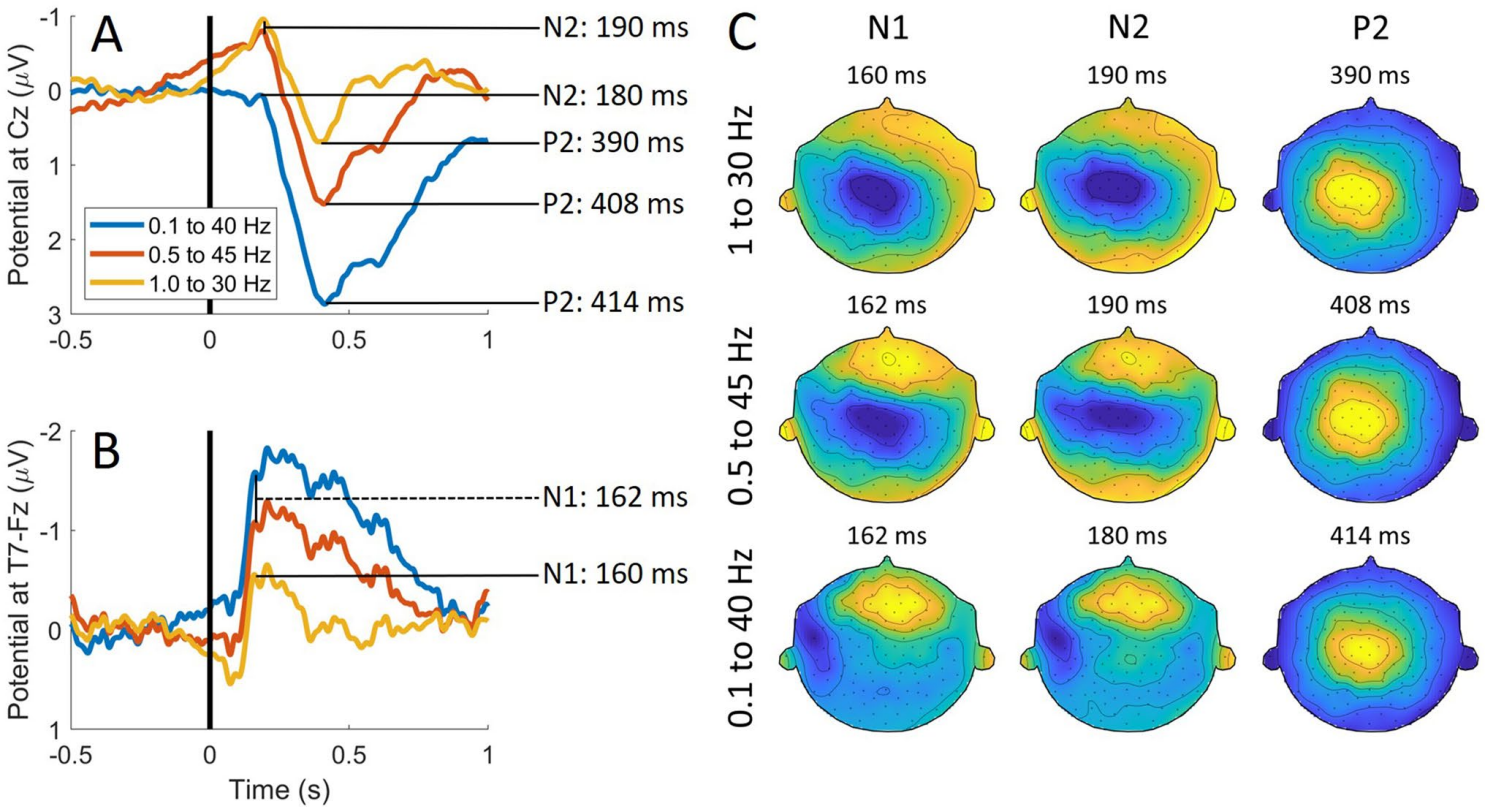
**a**, **b** Evoked potential at Cz (average reference) and T7-Fz band-pass filtered at 0.1 to 40 Hz, 0.5 to 45 Hz and 1 to 30 Hz, and latencies of the peaks in each of the EP waveforms. **c** Evoked potential topographies at the latencies of N1, N2 and P2

For comparison with previously observed EP waveforms in response to intra-epidermal stimulation in literature (Liang et al. 2016; Mouraux et al. 2014), EP waveforms at the same channels and with the same filters as in those studies are shown in Fig. 4a, b. The N1 was located at 160 ms (1 to 30 Hz) and 162 ms (0.1 to 40 Hz and 0.5 to 45 Hz) at T7-Fz. The N2, was located at 180 ms (0.1 to 40 Hz) and 190 ms (0.5 to 45 Hz and 1 to 30 Hz) at Cz. The P2 was located at 390 ms (1 to 30 Hz), 408 ms (0.5 to 45 Hz) and 414 ms (0.1 to 40 Hz).

Grand average EP topographies at these peak latencies are displayed in Fig. 4c. Topographies of the N1 and N2 were similar to each other for all filter settings, but differ per filter setting. The N1 and N2 both showed a central negative topography when using filters of 0.5 to 45 Hz and of 1 to 30 Hz. In contrast, the N1 and N2 showed a distinct contralateral topography when using filters of 0.1 to 40 Hz. The topography of the P2 was central and positive for all filter settings.

**Fig. 5:**
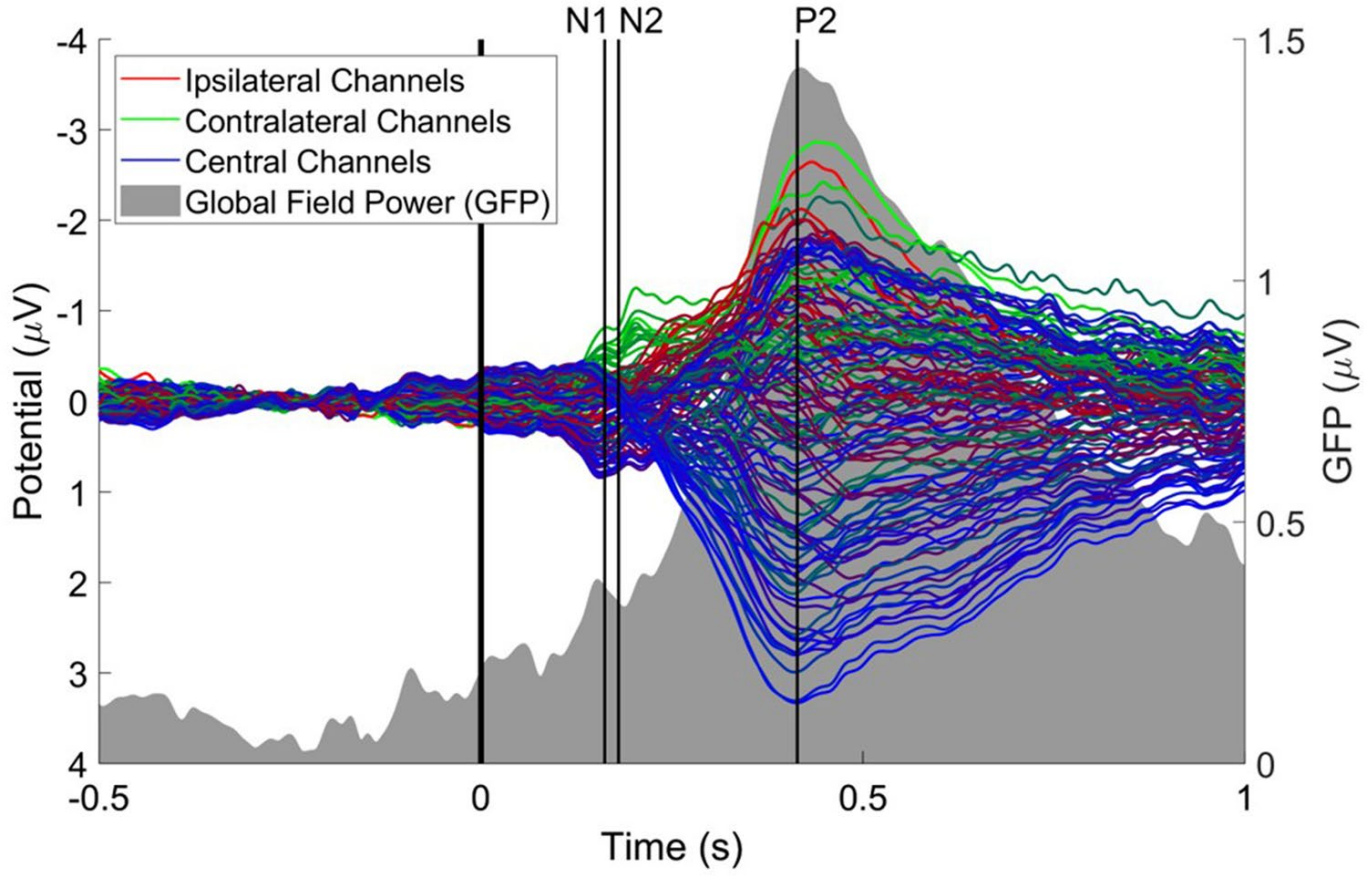
Butterfly plot of the grand average potential and global field power (GFP) of EEG channels in response to intra-epidermal stimuli around the nociceptive detection threshold (< 2 × NDT). The N1 appeared to coincide with an early peak of the GFP, while the P2 coincided with the maximum of the GFP. The N2 did not coincide with any peak in the GFP or in the butterfly plot

A butterfly plot of grand average EPs and the global field power (GFP) in response to the intra-epidermal electric stimuli is shown in Fig. 5. The global field power showed a major peak around the P2 and a minor peak around the N1, each corresponding to local maxima in a subset of channels. However, the N2 did not appear to coincide with any peak of global field power or of any subset of channels.

For each latency, the 12 channels with the largest positive and negative potentials are shown in Table 4. In addition, the SNR of each channel was computed, which in this case correlated strongly with the potential (i.e. the channels with the largest potential also have a large SNR). The derivation with the largest potential at each latency was found by subtracting the most negative channel(s) from the most positive channel or vice-versa depending on polarity. Based on these considerations, T7-F4 and CPz-A1A2 were selected for further investigation of the EP.

**Table 4:** Grand average electrode potential and SNR at N1, N2 and P2

	N1 Channel	Value (µV)	SNR	N2 Channel	Value (µV)	SNR	P2 Channel	Value (µV)	SNR
1	F4*	0.83	23.49	T7*	− 0.82	18.36	CPz**	3.33	185.57
2	T7*	− 0.82	18.22	F4*	0.77	21.76	CCP1h***	3.31	122.68
3	FFC2h***	0.81	17.41	F1**	0.75	14.41	CP1**	2.99	111.57
4	F2**	0.79	20.35	F2**	0.72	18.76	Cz*	2.80	97.37
5	Fz*	0.77	18.96	FFC2h***	0.72	15.42	CCP2h***	2.78	163.37
6	FFC4h***	0.71	15.24	TTP7h***	− 0.71	20.61	C1**	2.76	91.46
7	F1**	0.71	13.65	Fz*	0.70	17.12	A1*	− 2.74	36.57
8	AFF2***	0.69	19.38	AFz***	0.67	13.67	CPP2h***	2.64	111.59
9	FC2*	0.68	17.85	FT9***	− 0.63	12.16	CPP1h***	2.62	92.24
10	AFz***	0.68	13.88	AFF2***	0.63	17.81	CCP3h***	2.57	91.15
11	FT9***	− 0.64	12.24	FFC4h***	0.62	13.44	A2*	-2.56	30.74
12	TTP7h***	− 0.63	18.36	FC2*	0.59	15.44	CP2*	2.51	126.85

The electrodes with the largest potential values were also the electrodes with the largest SNR at N1 (F4) and P2 (CPz). Electrodes which are present in a regular 32-channel cap, 64-channel cap and 128-channel cap (10–5 system) are denoted with *, ** and *** respectively

**Fig. 6:**
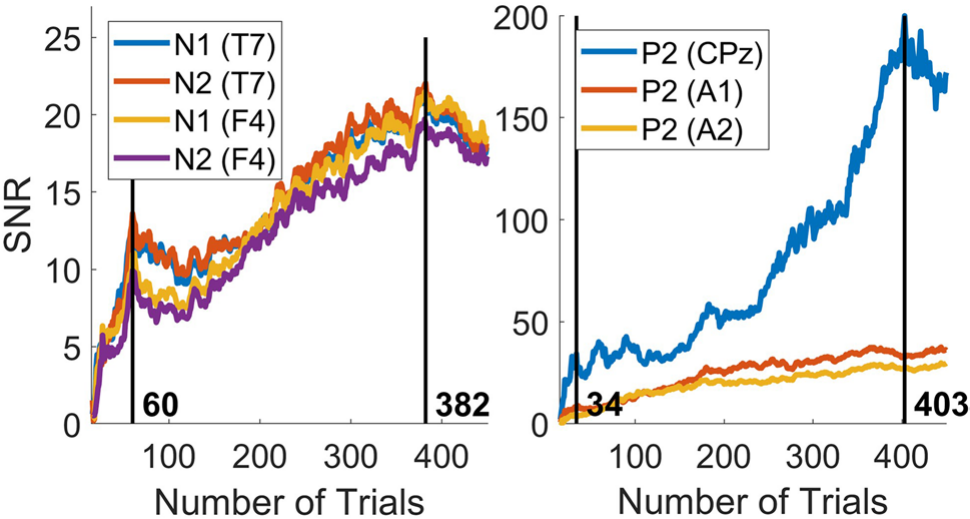
Signal-to-noise ratio (SNR) of the evoked potential in the grand average of the channels T7 and F4 (for N1 and N2), and CPz, A1 and A2 (for P2). For N1 and N2, there is an initial peak of the SNR after 60 trials, and a maximum at 382 trials. For P2, there is an initial peak of the SNR after 34 trials and a maximum at 403 trials

For the selected channels, the SNR is shown as a function of the number of trials in Fig. 6. For the N1 and N2, there is an initial peak of SNR after 60 trials. For the P2, there is an initial peak of the SNR after 34 trials. However, in both cases the SNR tends to increase with an increasing number of trials, with a maximum at 382 trials for the N1 and N2, and a maximum at 403 trials for the P2.

### Effect of Intra-Epidermal Stimulus Properties on Evoked Potential

**Fig. 7:**
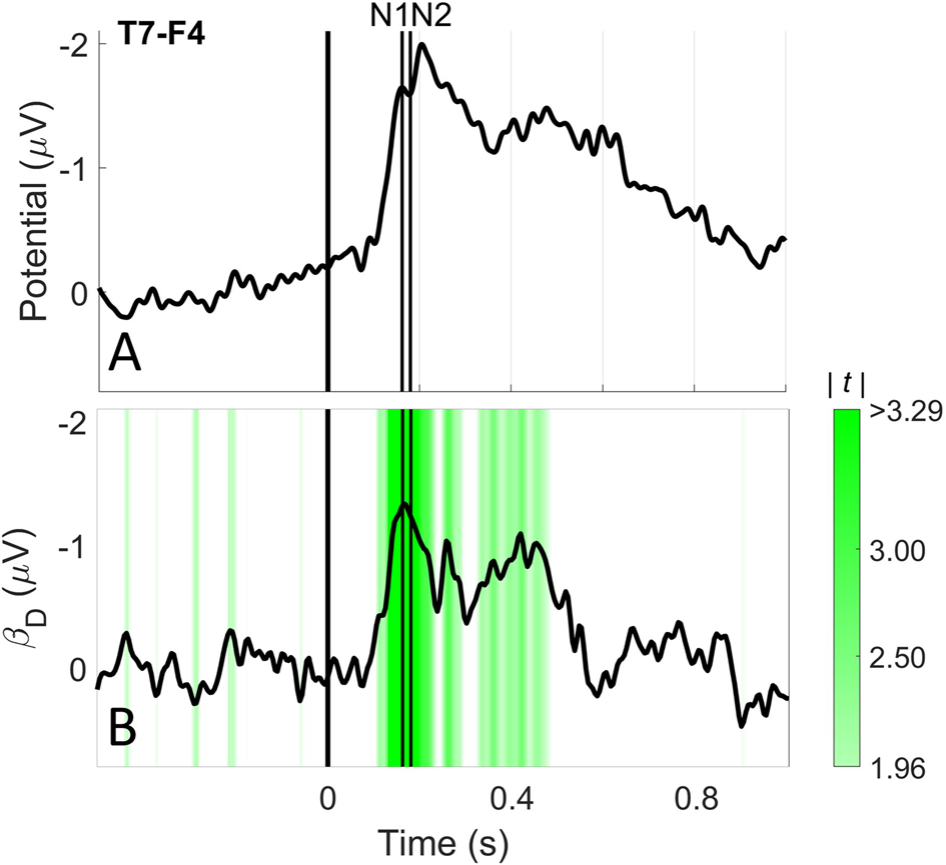
Grand average EP **a** and the effect of stimulus properties **b** at T7-F4. The corresponding t-values are shown in green on a scale of 1.96 (p = 0.05 with inf. DOF) to 3.29 (p = 0.001 with inf. DOF). At T7-F4 only stimulus detection had a significant effect on the EP at the N1 and N2 latencies

**Fig. 8:**
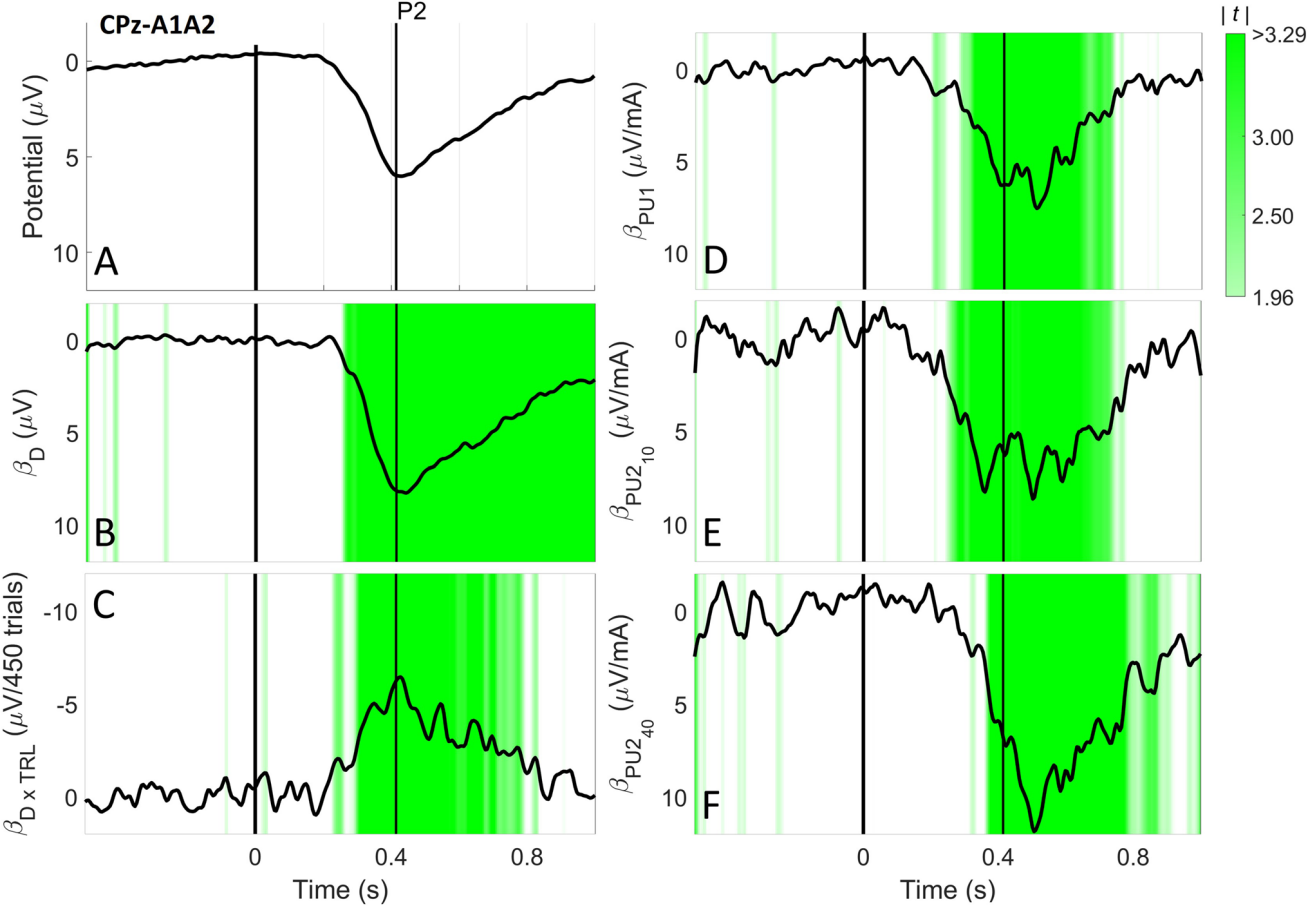
Grand average EP **a** and the effect of stimulus properties **b**–**f** at CPz-A1A2. The corresponding t-values are shown in green on a scale of 1.96 (p = 0.05 with inf. DOF) to 3.29 (p = 0.001 with inf. DOF). At CPz-A1A2 stimulus detection, the amplitude of each pulse and the interaction between detection and trial number had a significant effect on the EP at the P2 latency which lasted for several hundreds of milliseconds

The grand average EP at T7-F4 and CPz-A1A2 is shown in Figs. 7a and 8a respectively. At T7-F4 intra-epidermal stimuli elicited an early negative component with a peak around the N1 latency, followed by a small positive component. At CPz-A1A2, intra-epidermal stimuli elicited a clear positive component with a maximum close to the P2 latency.

The results of significance testing of stimulus properties at T7-F4 and CPz-A1A2 are shown in Table 5. At N1 and N2 only stimulus detection was significant. At P2 stimulus detection, each pulse amplitude and the interaction between detection and trial number were significant. For all significant effects, the effect sizes and *t*-values over time are displayed in Fig. 7b (for T7-F4) and 8B-F (for CPz-A1A2). For T7-F4, the negative effect of detection was mostly concentrated around the N1. For CPz-A1A2, the positive effect of each pulse amplitude started before the P2 and lasted for several hundred milliseconds. The interaction between detection and trial number had a negative effect during the same time range as the pulse amplitudes. Detection had a positive effect starting before the P2 and lasting until the end of the epoch.

**Table 5:** The effect sizes, confidence interval, t-values and corresponding p-values of the effect of stimulus properties on the EP at the latencies of N1 (T7-F4), N2 (T7-F4) and P2 (CPz-A1A2)

Stimulus property	Effect Size	T7-F4: N1			Effect Size	T7-F4: N2			Effect Size	CPz-A1A2: P2		
		95% CI	t	p		95% CI	t	p		95% CI	t	p
(Intercept)	0.61	[0.20, 1.02]	3.05	.005	0.62	[0.24, 0.99]	3.38	.002	− 3.55	[− 4.47, − 2.62]	− 7.91	** < .001**
Detection (D)												
Detected	− 1.30	[− 1.80, − 0.80]	− 5.32	** < .001**	− 1.29	[− 1.85, − 0.73]	− 4.69	** < .001**	8.07	[5.96, 10.18]	7.88	** < .001**
Pulse 1 (PU1)	− 0.41	[− 1.76 0.94]	− 0.63	.533	0.15	[− 1.19, 1.50]	0.23	.817	6.32	[3.24, 9.41]	4.27	** < .001**
Pulse 2, 10 ms IPI (PU2_10_)	− 2.00	[− 4.11 0.11]	− 1.86	.062	− 1.33	[− 3.66, 0.99]	− 1.21	.243	5.81	[2.75, 8.89]	3.71	** < .001**
Pulse 2, 40 ms IPI (PU2_40_)	0.95	[− 2.02 3.93]	0.66	.514	1.23	[− 1.48, 3.95]	0.94	.357	6.24	[3.15, 9.32]	4.12	** < .001**
Trial number (TRL)	1.26	[0.16 2.36]	2.29	.026	1.09	[− 0.02, 2.21]	1.99	.053	0.78	[− 1.00, 2.55]	0.89	.379
Trial number × Detection												
Trial number × Detected	− 0.18	[− 1.52 1.15]	− 0.27	.787	− 0.21	[− 1.69, 1.26]	− 0.29	.774	− 6.01	[− 8.47, − 3.56]	− 5.01	** < .001**

All effect sizes and confidence intervals were rescaled to physical units (Int.: -, D: µV/mA, PU1: µV/mA, PU2_10_: µV/mA, PU2_40_: µV/mA, TRL: µV/450 trials). At N1 and N2 only the detection was significant. At P2 all stimulus properties were significant, except the trial number

## Discussion

We have simultaneously assessed neurophysiological and psychophysical effects of nociceptive intra-epidermal stimulation using a method to simultaneously measure EPs and detection thresholds in response to multiple stimulus types. Preferential activation of nociceptive afferents was achieved by stimulating at intensities close to the detection thresholds, and excluding trials from EEG analysis if the stimulus was larger than twice the NDT (Mouraux et al. 2010). We aimed to confirm the presence, location and latency of EP components and to quantify the effect of intra-epidermal stimulus properties on those components.

### Evoked Potential in Response to Intra-Epidermal Stimuli

The EP waveforms observed in this study correspond with those observed in previous studies using intra-epidermal stimulation (Fig. 4a, b). Where an earlier study by Mouraux et al. (2014) observed a N1 component in response to intra-epidermal stimulation around a latency of 150 ms, we observed a N1 component at the same derivation (T7-Fz) and with the same filter settings around 160 ms. Furthermore, Mouraux et al. observed a N2 component at Cz in the range of 220–230 ms and another study by Liang et al. (2016) observed a much earlier N2 component at the same channel in the range of 130–150 ms. The observed N2 component in the current study occurs at the same channel and filter setting at a latency in between those estimates, around 190 ms. For both the N1 and N2 the largest positive and negative potential values and SNR were found at F4 and T7 respectively. The SNR of both components at T7 and F4 was shown to increase with respect to the number of trials included in the grand average. However, after a steep increase of the SNR within the first 60 trials, the improvement of SNR flattens, potentially due to habituation affects and loss of attention.

The observed latencies of the P2 at Cz of 390 and 408 ms, are slightly later than those observed by Liang et al. (290–330 ms) and Mouraux et al. (360–370 ms) respectively. Although the observed amplitude is much lower than in studies using heat pulses (Miyazaki et al. 1994; Treede et al. 1988), it is comparable to earlier nociceptive EP studies using intra-epidermal stimulation near the detection threshold (Mouraux et al. 2010; Van der Lubbe et al. 2017), indicating that this difference in potential might be due to stimulus intensity. The potential values in Table 4 show that during the observed P2, the largest positive potential value (and SNR) could be found at CPz and the lowest negative potential value (and SNR) could be found at A1 followed by A2. The SNR of the P2 at CPz, A1 and A2 improved the most within the first 34 trials, but kept increasing steeply until reaching a maximum close to the end of the experiment at 403 trials.

Although the observed EP waveform corresponds to previous studies using similar derivations and filter setting, it is clear from Fig. 4 that filter settings have a profound influence on the EP waveform and topography. While the choice of a larger cutoff frequency for the high-pass filter enhances the N2 component at Cz, it strongly decreases amplitude of the P2. Furthermore, the filter setting has a profound influence on the observed topographies of N1 and N2. While the topographies of N1 and N2 in this study show a maximum around the vertex with a high-pass filter of 0.5 or 1 Hz, both topographies have a maximum contralateral to stimulation when using a high-pass filter of 0.1 Hz. In this work, we chose to minimize high-pass filter signal distortion, by choosing a relatively low high-pass filter cutoff frequency of 0.1 Hz (Acunzo et al. 2012).

When using a low high-pass filter cutoff frequency, the P2 amplitude is increased, but the N2 amplitude is severely reduced. Using this filter setting the observed global field power showed peaks only around the N1 latency and the P2 latency. Topographies of the N1 and N2 are almost identical, with a maximum at T7 and a minimum at F4. As such, it is questionable whether N1 and N2 identified in this study really represent independent components of brain activity. In the current study, it appears that the N2 rather arose from high-pass filter settings rather than physiological activity, as the high-pass filter essentially works as a signal differentiator resulting in peaks of opposite polarity before occurrence of the true effect, i.e. the P2 (Tanner et al. 2015).

### Effect of Intra-Epidermal Stimulus Properties on Detection Probability

Intra-epidermal electric stimuli directly activate superficial afferents in the skin, rather than activating skin receptors (Inui and Kakigi 2012; Inui et al. 2002). As this signal is transduced by the relatively slow Aδ-fibers, rather than the fast Aβ-fibers, the reaction times to this type of stimulation are usually increased with respect to conventional transcutaneous electric stimulation. Correspondingly, the average reaction time in this study (546 ± 161 ms) was markedly later than previously observed reaction times (Mouraux et al. 2010) to transcutaneous stimulation (283 ± 47 ms) and intra-epidermal stimulation at twice the perceptual threshold (374 ± 51 ms), but similar to laser stimulation (504 ± 105 ms). As such, reaction times suggest preferential recruitment of Aδ-fibers.

Increasing the pulse amplitude directly enlarges the area of recruitment of peripheral afferent nerve fibers (Poulsen et al. 2020). Increasing the number of pulses results in the generation of more action potentials. Central synaptic summation of these action potentials occurs in the spinal cord, where facilitatory or inhibitory effects could occur depending on the inter-pulse interval (Zucker and Regehr 2002). Nociceptive processing adapts to repeated stimulus application, leading to a habituation of neurophysiological (Christmann et al. 2007) and psychophysical (May et al. 2012) responses. One of the aims was to probe each of these mechanisms by varying pulse amplitudes, the number of pulses and the inter-pulse interval of the applied stimuli. We started out by formulating and comparing multiple statistical models to study the effect of these stimulus properties. This included functional models explaining detection probability and EEG in terms of pulse amplitudes and trial number. It was found in Table 1 that both functional models (A and E) have the lowest BIC, indicating that among the tested models these models are the closest approximation of the true physiological behavior.

We found that nociceptive stimulus detection behaves according to theory, where the pulse amplitudes and trial number have a significant effect on the detection probability. The effects of pulse amplitudes observed in this study lie within the confidence interval reported in the earlier technical demonstration of the method (van den Berg et al. 2020). As was expected based on the larger recruitment of peripheral afferents, the detection probability increased with increasing pulse amplitudes. Furthermore, we saw that addition of a second pulse with either 10 ms or 40 ms inter-pulse interval leads to a significant increase of detection probability (Fig. 3; Table 2) similar to earlier observations by Doll et al. (2016a, b). This results in significantly lower detection thresholds and significantly steeper slopes for these double pulse stimuli (Table 3). However, there is no difference between double-pulse detection thresholds and slopes dependent on the inter-pulse interval, which was either 10 or 40 ms. Experiments in humans subjects measuring the compound sensory action potential in response to paired pulses indicate that peripheral sensory nerve fibers remain superexcitable up to approximately 20 ms after the first pulse and remain subexcitable from 20 to 100 ms after the stimulus (Kiernan et al. 1996). If these findings also hold for nociceptive afferents, one would expect a higher detection probability and lower threshold for double-pulse stimuli with an inter-pulse interval of 10 ms than for double-pulse stimuli with an inter-pulse interval of 40 ms. It turns the effect of adding a second-pulse is similar regardless of the inter-pulse interval, resulting in similar detection thresholds for both stimulus types. As such, the effects of peripheral super- and subexcitability appear to be canceled out by a stronger central mechanism, such as the central temporal summation of both pulses (Zucker and Regehr 2002).

The detection probability decreases over the number of trials, resulting in an increase of the detection threshold in Fig. 3. In earlier studies, this effect was also found significant, but had a larger effect size (Doll et al. 2016a, b; van den Berg et al. 2020). Altered habituation appears to play an important role in several types of chronic pain syndromes, and is therefore an important phenomenon to observe when assessing nociceptive processing (Agostinho et al. 2009; Rodriguez-Raecke et al. 2014; Valeriani et al. 2003). Nevertheless, the neurophysiological mechanisms of this effect remain unknown, and this effect might be attributed to either an altered task performance, a shift of attention, learning or neuroplasticity.

### Effect of Intra-Epidermal Stimulus Properties on Evoked Potential

The effect of stimulus properties on the EP was shown in Table 5. Based on Figs. 7b and 8b–f we could also observe at which latencies these effect sizes were largest. We did not find that the lateral potential at T7-F4 behaves according to the theory mentioned earlier. Instead, it was only significantly modulated by detection with its major effect size around the latency of N1. As such, we did not observe any significant encoding of physical properties of the stimulus (i.e. the pulse amplitudes) in the N1 or N2. It remains unknown if this absence of the effect of stimulus properties on lateral EP components is because these components do not encode any physical properties of a stimulus, or simply because the N1 and N2 are relatively small signals and easily obscured by background noise.

The potential at CPz-A1A2 around at P2 latency was not only modulated by detection, but also by the pulse amplitudes and the interaction between detection and trial number. The latter observation is consistent with earlier reports in literature, which show that the P2 represents multi-modal activity dependent on stimulus salience (Iannetti et al. 2008; Legrain et al. 2011). As the pulse amplitudes and the trial number influence stimulus salience through the mechanisms discussed in last section, these properties were also expected to affect the P2. We saw that the contribution of the second pulse is equal or even larger than the contribution of the first pulse to the P2. This corresponds to the observation in last section, where the detection threshold is lowered by the addition of a second pulse and the effect size of both types of second pulse is actually larger than the effect size of the first pulse amplitude. As such, the observed increase in P2 amplitude is likely to be associated with similar facilitatory mechanisms as discussed in “Effect of Intra-Epidermal Stimulus Properties on Detection Probability” section.

We also observed a significant effect of the interaction between detection and trial number on the P2 amplitude. As such, the P2 decreases with respect to trial number, but only in response to detected stimuli. Measuring this habituating behavior can be used to assess altered nociceptive processing. A decreased habituation of P2 amplitude over time or over the amount of repeated stimuli has been related to chronic pain in earlier literature in patients with migraine (Valeriani et al. 2003), chronic low back pain (Vossen et al. 2015) and fibromyalgia (de Tommaso et al. 2011).

## Conclusion

After a technical demonstration of combined threshold tracking and EP acquisition in an earlier study (van den Berg et al. 2020), we started this study to determine (1) which EP components can be observed during this procedure, (2) at which scalp locations these components are best observed and (3) to quantify the effect of stimulus properties on these components and detection thresholds in healthy subjects. We found that an N1 and N2 component can be observed with a maximum positive and negative potential at F4 and T7 respectively. The P2 component can be observed with maximum positive and negative potentials at CPz and A1 respectively. The P2 has a similar latency and topography regardless of filter settings. However, the N1 and N2 waveform and topography are heavily affected by the high-pass cutoff frequency. Using a larger cutoff frequency enhanced the N2 and shifted the topographies of N1 and N2 from contralateral to central, suggesting that the observed N2 could be an artifactual effect of high-pass filtering. Statistical analysis showed that the N1 and N2 components observed in this experiment mainly influence by stimulus detection, while the P2 as well as the detection probability of a stimulus are also significantly influenced by stimulus properties such as the pulse amplitudes and the trial number.

Measuring the effects of intra-epidermal stimulus properties on the detection threshold and the evoked potential simultaneously provides a way to measure brain activation and pain perception in response to a well-defined nociceptive input. The results in this study demonstrate that the various steps of processing of a nociceptive stimulus, including peripheral nerve fiber recruitment, central synaptic summation, and habituation to a repeated stimulus are reflected by the detection thresholds as well as the EP.

## Data Availability

A limited dataset of the experiments reported here is available on request.

## Code Availability

Code required to perform the analyses reported here is available on request.
